# High-dimensional Cytometry (ExCYT) and Mass Spectrometry of Myeloid Infiltrate in Clinically Localized Clear Cell Renal Cell Carcinoma Identifies Novel Potential Myeloid Targets for Immunotherapy

**DOI:** 10.1074/mcp.RA120.002049

**Published:** 2020-07-31

**Authors:** Debebe Theodros, Benjamin M. Murter, John-William Sidhom, Thomas R. Nirschl, David J. Clark, LiJun Chen, Ada J. Tam, Richard L. Blosser, Zeyad R. Schwen, Michael H. Johnson, Phillip M. Pierorazio, Hui Zhang, Sudipto Ganguly, Drew M. Pardoll, Jelani C. Zarif

**Affiliations:** 1 Medical Scientist Training Program, Johns Hopkins University School of Medicine, Baltimore, Maryland, USA; 2 Graduate Program in Immunology, Johns Hopkins University School of Medicine, Baltimore, Maryland, USA; 3 Bloomberg-Kimmel Institute for Immunotherapy, Johns Hopkins University School of Medicine, Baltimore, Maryland, USA; 4 Department of Biomedical Engineering, Johns Hopkins University School of Medicine, Baltimore, Maryland, USA; 5 Pathobiology Graduate Program, Johns Hopkins University School of Medicine, Baltimore, Maryland, USA; 6 Department of Oncology, Johns Hopkins School of Medicine and The Sidney Kimmel Comprehensive Cancer Center, Baltimore, Maryland, USA; 7 Department of Pathology, The Johns Hopkins University School of Medicine, Baltimore, Maryland, USA; 8 The James Buchanan Brady Urological Institute, Johns Hopkins University School of Medicine, Baltimore, Maryland, USA

**Keywords:** immunotherapy, monocytes, macrophages, peripheral blood mononuclear cells, tumor microenvironment, ExCYT, kidney cancer, immunology, clinical proteomics, mass spectrometry, clear cell renal cell carcinoma

## Abstract

Renal Cell Carcinoma (RCC) is one of the most commonly diagnosed cancers worldwide with research efforts dramatically improving understanding of the biology of the disease. To investigate the role of the immune system in treatment-naïve clear cell Renal Cell Carcinoma (ccRCC), we interrogated the immune infiltrate in patient-matched ccRCC tumor samples, benign normal adjacent tissue (NAT) and peripheral blood mononuclear cells (PBMCs isolated from whole blood, focusing our attention on the myeloid cell infiltrate. Using flow cytometric, MS, and ExCYT analysis, we discovered unique myeloid populations in PBMCs across patient samples. Furthermore, normal adjacent tissues and ccRCC tissues contained numerous myeloid populations with a unique signature for both tissues. Enrichment of the immune cell (CD45^+^) fraction and subsequent gene expression analysis revealed a number of myeloid-related genes that were differentially expressed. These data provide evidence, for the first time, of an immunosuppressive and pro-tumorigenic role of myeloid cells in early, clinically localized ccRCC. The identification of a number of immune proteins for therapeutic targeting provides a rationale for investigation into the potential efficacy of earlier intervention with single-agent or combination immunotherapy for ccRCC.

## Abstract (precis)

Although the focus of the role of cancer immunotherapy has been in advanced disease states, we sought to investigate changes to the immune infiltrate of early, clinically localized clear cell Renal Cell Carcinoma (ccRCC). Using orthogonal approaches including Mass Spectrometry on immune cell infiltrates, we report numerous alterations that provide new insight into the biology of treatment-naïve ccRCC and identification of novel targets that may prove to be clinically impactful.

## Graphical Abstract

**Figure:**
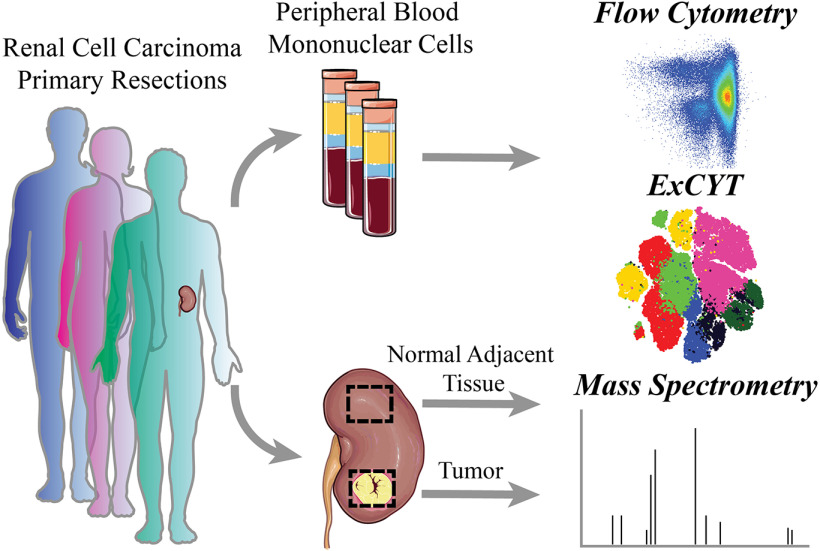

- Using ExCYT, genomics, and Mass Spectrometry, we were able to uncover immune cell marker alterations that provide new insight into the biology of early stage ccRCC.
- Among the CD45^+^ population, we observed a high level of myeloid cell infiltration in treatment-naïve ccRCC tissues.

Renal cell carcinoma (RCC) is one of the most commonly diagnosed cancers with numerous molecular and histological features identified leading to tailored therapeutic approaches (1, 2). Standard of care options for clinically localized RCC include partial and radical nephrectomy, active surveillance, and thermal ablation (3). However, up to 30% of patients progress and develop metastatic disease that is associated with a high mortality rate (4–7). We interrogated the immune infiltrate of seven patient-matched clear cell Renal Cell Carcinoma (ccRCC) tumors, normal adjacent tissue (NAT) and peripheral blood mononuclear cells (PBMCs) isolated from whole blood. Flow cytometric, MS, and ExCYT analysis revealed unique myeloid populations in PBMCs across patients. NAT and ccRCC tissues contained numerous myeloid populations with unique signatures for both tissues. Immune cell (CD45^+^) enrichment and gene expression analysis revealed numerous differentially expressed myeloid genes. These data provide evidence of an immunosuppressive and pro-tumorigenic role of myeloid cells in clinically localized ccRCC. Identification of numerous novel immune proteins for therapeutic targeting may delay progression of disease and provides a rationale for the investigation of early intervention with single-agent or combination immunotherapy for ccRCC.

Mounting evidence implicates a role of the immune system in ccRCC that depends on stage of disease. Furthermore, recent analyses reveal a diverse and heterogeneous immune cell infiltrate in clear cell RCC (ccRCC) composed of both lymphocytes and myeloid cells (8–11). Genetic alterations that influence susceptibility to immunotherapy continue to be identified, further solidifying the impactful role of the immune system in ccRCC (12). Although a growing number of studies continue to investigate the immune cell composition in metastatic or late-stage disease, few studies interrogate changes to immune cell infiltrate and gene expression in early stage disease. One study identified changes in the immune compartment within the peripheral blood of patients in association with disease severity (13). A detailed analysis of the immune cell infiltrate in early-stage, clinically localized disease could provide insight into the mechanisms by which ccRCC evades the immune system and progresses to advanced disease. In addition, knowledge of disease initiation and pro-tumorigenic signals expressed by the immune system provide therapeutic opportunities.

We sought to explore the myeloid cell infiltrate in ccRCC tumor microenvironment (TME), 'normal' adjacent tissue (NAT), and peripheral blood mononuclear cells (PBMCs) by flow cytometry and ExCYT (14). Using MS and flow cytometry, our results reveal differences in myeloid populations found in the PBMCs across patients, and unique myeloid populations between NAT and ccRCC tissues. Using Nanostring Technology, we interrogated the expression of 700+ myeloid-related genes from CD45^+^ enriched fractions from tumor and NAT with top hits validated by reverse transcription quantitative PCR (RT-qPCR). Furthermore, analysis of CD45^+^ enriched fractions revealed differentially expressed pathways utilized by immune cells and nonimmune cells in the tumor microenvironment as well as NAT. Thus, we identified early changes in the host myeloid cell compartment that may provide opportunities for new treatment strategies.

## MATERIALS AND METHODS

### 

#### 

##### Tissue Dissociation

Consented tissue samples were obtained from JHU Pathology under an institution approved IRB protocol. Following resection, tissues were submerged in DMEM + 10% Heat-Inactivated Fetal Bovine Serine (HiFBS, GE) on ice. Samples were minced and dissociated using the Human Tumor Dissociation Kit (cat #: 130-095-929) and gentleMACS Octo Dissociator (cat #: 130-095-937, Miltenyi). Single-cell suspension was subjected to ∼3 ml of ACK red cell lysis buffer (Quality Biological cat #: 118-156-721) for ∼3 min then brought to 40 ml volume with sterile 1 × Phosphate-Buffered Saline (Quality Biological cat #: 114-058-101) and spun down at 1300 RPM for 5 min at 4 °C. Cells were re-suspended in 1 ml of sterile 1 × PBS and counted for downstream applications.

##### Isolation of Peripheral Blood Mononuclear Cells

Whole blood was diluted in 1 × PBS at a 1:1 volume level to a maximum volume of 35 ml. A glass pipette was then used to underlay samples with 15 ml of Ficoll (GE Healthcare cat #: 17-1440-02). Samples were spun at 2200 RPM for 22 min at 20 °C with no brake. Cells at the interface were harvested and washed with 40 ml PBS twice. Cells were then subjected to ∼ 3 ml of ACK red cell lysis buffer for ∼3 min then, brought to 40 ml volume with 1 × PBS and spun at 1300 RPM for 5 min at 4 °C. Cells were re-suspended in 1 ml of sterile 1 × PBS and counted for downstream applications.

##### Flow Cytometry

Patient-matched PBMCs, NAT, and Tumor cells were rapidly thawed in DMEM + 10% HFBS, spun down at 1300 RPM for 5 min and then re-suspended in PBS, counted, and plated into a 96-well u-bottom plate. Viability dye staining was performed using the Invitrogen Molecular Probes LIVE/DEAD Fixable Dead Cell Stains (cat #: L34957 ThermoFisher Scientific) at 1:1000 for 15 min at room temperature. Cells were brought up to volume (200 µL) with PBS, spun down, and re-suspended in Fc block (1:50, cat #: 564765 BD Human anti-CD16/32 block, cat #: 562162 BD Human anti-CD64 block) at 4 °C for 15 min. Surface marker staining was performed and cells were also stained in True-Stain Monocyte Blocker (Biolegend). Surface markers used include CD11b-BV650 1:100, CD15-BV786 1:250, CD33-BV605 1:200, PD-L1-BV421 1:250, CD14-APC-H7 1:50, CD45-AF700 1:150, HLA-DR-PE-CF594 1:500, Lineage (CD3, CD19 CD56)-FITC 1:25, CD16-PeCy7 1:50. Cells were washed in MACS buffer (1 × PBS, 0.5% BSA, 2 mm EDTA) and run on the BD FACS Celesta Flow Cytometer. Flow data were analyzed using FlowJo Software version 10.4.2.

##### ExCYT Analysis

To perform a high-dimensional analysis of the flow cytometry data, we used ExCYT, a new available software to perform dimensionality reductions and clustering analysis of the data (https://github.com/sidhomj/ExCYT) (14). For all samples, we first gated cells by FSC/SSC, single cells by FSC-A/SSC-A, L/D-, CD45^+^, Lymphocyte^−^ before randomly sampling 10,000 cells/sample for downstream analysis. We performed t-SNE analysis for creating visualizations and applied a Gaussian Mixture Model (GMM) via Expectation Maximization to apply clustering solutions. We then used the software package to visualize these clustering solutions via high-dimensional flow plots (14).

##### CD45^+^ Enrichment, RNA Extraction, cDNA Generation, qRT-PCR

CD45^+^ enrichment was performed using the Miltenyi Human CD45^+^ Enrichment Kit (cat #: 130-045-801). RNA from CD45^+^ enriched cell fractions was isolated using the commercially available Qiagen RNeasy mini kit and quantified using the NanoDrop. cDNA was generated using the High-Capacity RNA-to-cDNA Kit (Applied Biosystems catalog #4368814) according to manufacturer's protocol. 12.5 ng of cDNA was loaded per reaction onto a TaqMan Array 96 – Well FAST Plate (Applied Biosystems), as well as TaqMan probes [CSF-1R: ThermoFisher Scientific Hs00911250_m1, MSR1: ThermoFisher Scientific Hs00234007_m1, CX3CR1: ThermoFisher Hs01922583_s1. All plates were run on a StepOnePlus Real-Time PCR System machine (Applied Biosystems). Fold Expression was calculated as log_2_^^-ΔΔCT^.

##### Nanostring Gene Expression Analysis

RNA was isolated from CD45^+^ cells enriched from both normal and ccRCC samples as described above. The nCounter GX human Immunology V2 Kit (NanoString Technologies) was used to measure the expression of >700 human genes in the RNA of these samples. Following hybridization, transcripts were quantitated using the nCounter Digital Analyzer. Samples were run at the Johns Hopkins Deep Sequencing & Microarray Core facility. To correct for background levels, the highest negative control value for each sample was subtracted from each count value of that sample. Following background subtraction, any negative count values were considered as 0. The geometric mean of 5 housekeeping genes provided by the company panel was calculated and used to normalize expression values. Fifty cross-reactive genes were removed prior to analyses of the data set. Nonexpressed genes were defined as < 100 relative RNA counts and below four times the standard deviation in all samples.

##### Experimental Design and Statistical Rationale

A total of ten samples were analyzed by MS-based proteomics. CD45^+^ cells from tumor and normal adjacent tissue (NAT) regions, CD45- cells from tumor and NAT regions, and PBMC cell populations were derived from two patient populations, representative of two biological replicates for each cell population. We prioritized comparison of the proteomes of CD45^+^ cell populations derived from tumor and NAT regions, with proteins quantified across all four samples considered for downstream bioinformatic analysis. Protein abundance was determined using label free quantitation (LFQ) in the MaxQuant software suite, with proteins categorized as overexpressed (log_2_ fold change > 1.0) subjected to overrepresentation enrichment analysis (ORA). Pathways were considered statistically significant with *p*-values and false discovery rates (FDR) < 0.05.

##### In-Solution Digestion

Individual cell pellets were processed as previously described with some modifications (15). In brief, pellets were resuspended in lysis buffer (8M urea, 75 mm NaCl, 50 mm Tris, pH 8.0, 1 mm EDTA, 2 µg/ml aprotinin, 10 µg. Ml leupeptin, 1 mm PMSF, 10 mm NaF, phosphatase inhibitor mixture 2 and phosphatase inhibitor mixture 3 (1:100 dilution), and 20 μm PUGNAc), and homogenized via vortexing Samples were centrifuged at 20,000 × *g* and the resulting supernatant retained, and protein concentration was measured via BCA Protein Assay. Protein lysates were subjected to reduction with 5 mm 1,4-Dithiothreitol for 30 min at RT, followed by alkylation with 10 mm iodoacetamide for 45 min at RT in the dark. Urea concentration was reduced < 2M using 50 mm Tris-HCl, pH 8.0, and LysC/Trypsin was added in an enzyme to substrate ratio of 1:40 and samples incubated overnight at 37 °C. The generated peptides were acidified to a final concentration of 1% formic acid, subjected to clean-up using C-18 SepPak columns (Waters), and then dried.

##### Nano-ESI-LC–MS/MS Analysis

One µg of peptide was separated using Easy nLC 1200 UHPLC system (Thermo Scientific) on an in-house packed 20 cm × 75 μm diameter C18 column (1.9 μm Reprosil-Pur C18-AQ beads (Dr. Maisch GmbH); Picofrit 10 μm opening (New Objective)). The column was heated to 50 °C using a column heater (Phoenix-ST). The flow rate was 0.200 μl/min with 0.1% formic acid and 2% acetonitrile in water (A) and 0.1% formic acid, 90% acetonitrile (B). The peptides were separated with a 6-30% B gradient in 84 min and analyzed using the Thermo Fusion Lumos mass spectrometer (Thermo Scientific). Parameters were as followed MS1: resolution – 60,000, mass range – 350 to 1800 m/z, RF Lens – 30%, AGC Target 4.0e^5^, Max IT – 50 ms, charge state include - 2-6, dynamic exclusion – 45 s, top 20 ions selected for MS2; MS2: resolution – 15,000, high-energy collision dissociation activation energy (HCD) – 34 (nonTMT-labeled samples) or 37 (TMT-labeled samples), isolation width (m/z) – 0.7, AGC Target – 5.0e^4^.

##### Protein Identification, Quantification, and Pathway Analysis

The resulting MS/MS spectra were searched against UniprotKB Swiss-Prot human protein database (version June 2017; 20,192 reviewed sequences) using MaxQuant and the integrated Andromeda search engine (version 1.5.3.30), using many of the default search parameter settings (16–18). Enzyme specificity was set to Trypsin/P, minimal peptide length of 7 amino acids, and allowing for two missed cleavages. Variable modifications included methionine oxidation and N-terminal protein acetylation, and fixed modification of carbamidomethylation on cysteine. Default MaxQuant Orbitrap search settings were used, included mass tolerance of 20 ppm for precursor and fragment ions, and a maximum false discovery rate (FDR) of 1% for peptide spectral matched (PSMs) and proteins. Data were further processed using the label-free quantitation (LFQ) parameter, with a minimum of one peptide for protein level quantitation. The MS proteomics data have been deposited to the ProteomeXchange Consortium via the PRIDE partner repository (19)with the data set identifier PXD014370. In addition, MaxQuant search results were uploaded to MS-Viewer (20). With the data set identifier dztdd1npww. Search results were imported into Perseus (version 1.5.4.1) and further processed (21). Proteins quantified in both replicates from each of the conditions were considered for downstream analysis. LFQ Intensities were transformed to log2 values and proteins categorized as overexpressed (log2 fold change > 1.0) were subjected to overrepresentation enrichment analysis (ORA) using the bioinformatics tool, WebGestalt (22, 23), and mapped to REACTOME and KEGG pathways.

##### Statistical Analysis

Statistical analyses were performed using GraphPad (Prism 6) or ExCYT. Parametric Student's *t* test (two-way groups or unpaired) were performed. Analyses were considered significant as follows: * *p* ≤ 0.05, ** *p* ≤ 0.01, *** *p* ≤ 0.001, **** *p* ≤ 0.0001. Flow cytometric samples were performed as one experiment following banking of all samples and data represented as cumulative. NanoString experiment performed as one experiment and data represented as cumulative. qRT-PCR experiments performed as multiple experiments and cumulative data plotted. Additional details present within each figure legend.

## RESULTS

### 

#### 

##### Flow Cytometric and ExCYT Analysis Reveal Myeloid Cell Infiltration in Clinically Localized ccRCC

**Fig. 1.**
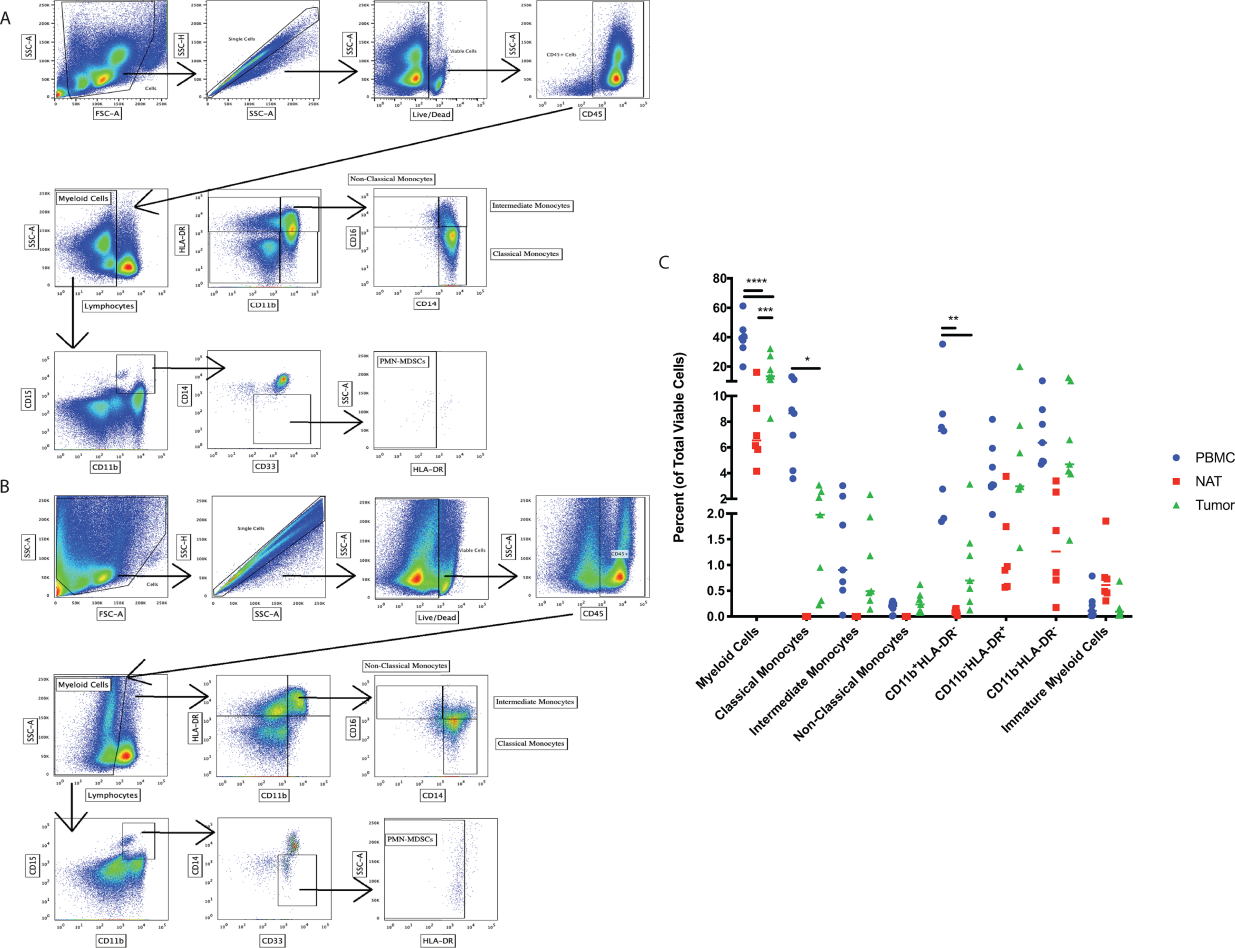
**The Myeloid Population Infiltrate in localized ccRCC.** Flow cytometry data revealing gating schemes used similarly for all tissues (Patient-matched PBMCs, NAT, and tumor) with depiction of a representative series of flow plots for PBMCs (*A*) and tumor (*B*). The percentage of various myeloid cell populations based upon the parent gate for PBMCs (*blue*), NAT (*red*), and tumor (*green*) are shown (*C*).

To investigate myeloid cell heterogeneity and infiltrate, we obtained patient-matched PBMCs, NAT, and ccRCC and stained for myeloid markers to analyze by flow cytometry (Fig. 1*A*–1*B*; supplemental Table S1, supplemental Fig. S1). We identified differences between tissue compartments across a number of myeloid cell populations (Fig. 1*C*). Interestingly, the tumor microenvironment (TME) contained significantly greater total myeloid cells by percent of total viable cells, relative to the patient-matched NAT. Particularly, there was a significant reduction in the numbers of classical (CD14^+^CD16^−^) monocytes in the TME relative to the blood with no significant difference in the percentage of intermediate (CD14^+^CD16^+^) and nonclassical (CD14^-^CD16^+^) monocytes between the TME and blood. Interestingly, there were fewer CD11b^+^HLA-DR^−^ cells in the TME and NAT relative to the blood.

**Fig. 2.**
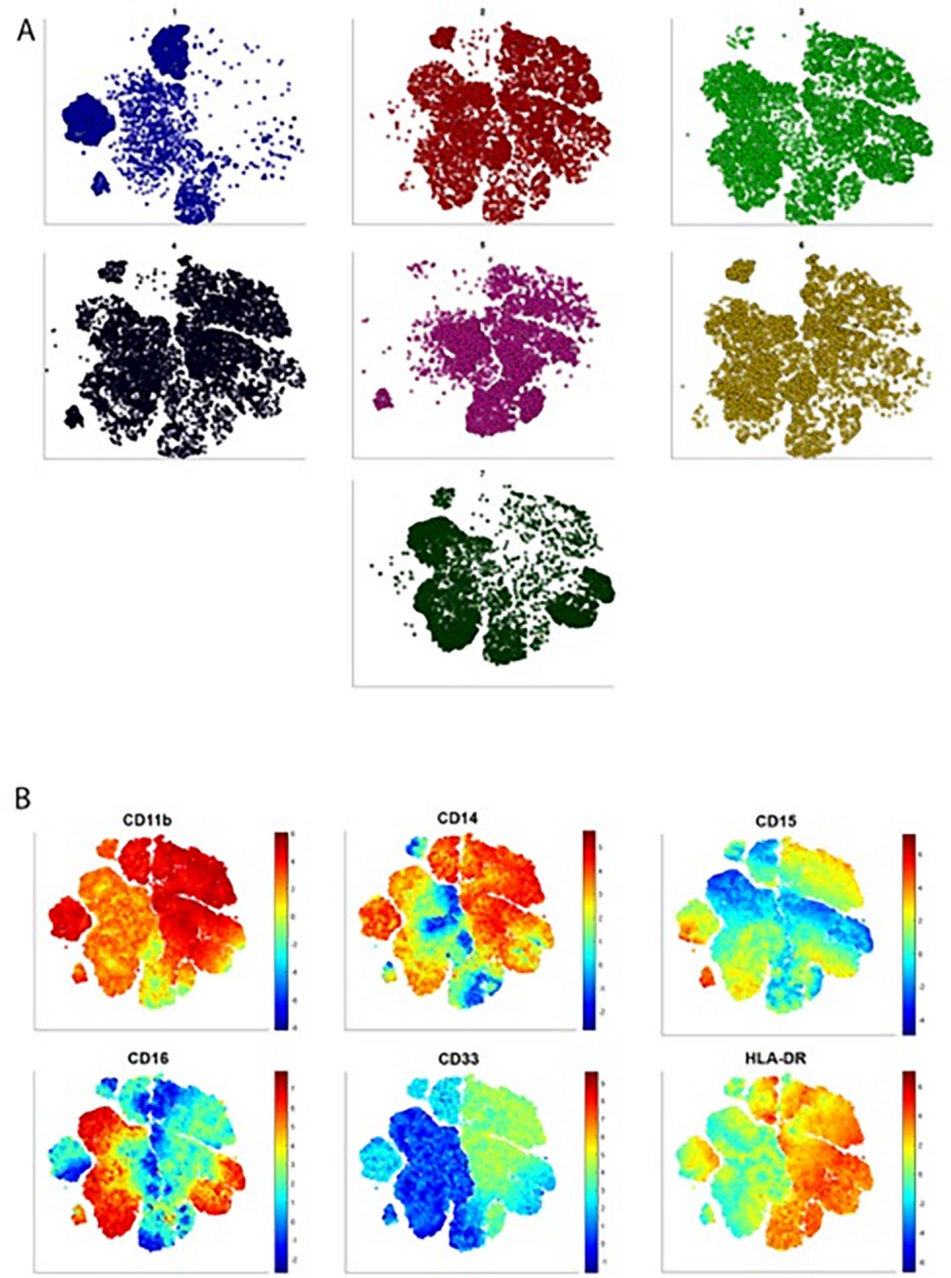
**ExCYT Analysis Identifies Unique Myeloid Signatures in the Blood.** (*A*) Each t-SNE plot has been generated from the PBMC flow cytometry data for each individual patient. (*B*) Each t-SNE plot represents pooled flow cytometry data from all 7 patient PBMC flow data. The PBMC flow cytometry data were then gated on viable cells CD45^+^ lymphocyte- and the relative expression of each of 6 myeloid markers (CD11b, CD14, CD15, CD16, CD33, HLA-DR) is expressed with red indicating higher levels of expression and blue indicating lower levels of expression.

Next, we sought to investigate the potentially unique clusters expressed in the PBMC, NAT, or TME in an unsupervised manner (Fig. 2*A*, supplemental Fig. S1*A*, markers listed in method section). To enable relatively facile and detailed analysis of the flow cytometry data, we developed a novel graphic user interface to facilitate analysis of the high-dimensional data for analysis in supervised and unsupervised manners (14). Representation of the PBMC flow cytometry data by t-SNE, an unsupervised dimensionality reduction method to visualize high-dimensional data, revealed differences between patients that may not have been appreciated by conventional gating strategies. Interestingly, PBMC data from patient 1 exhibited a unique cell population characterized by high expression of CD11b and CD14, intermediate to low expression of HLA-DR and low expression CD15, CD16, CD33 (Fig. 2*B*). This population was unique to patient 1 and absent in the other 6 patients. Patients 2, 4, 6, and 7 (and to a small extent, patients 3 and 5) exhibited a cell population best characterized as expressing high levels of CD11b, intermediate/low levels of HLA-DR and low levels of CD14, CD15, CD16 and CD33. Patients 3 and 7 exhibited a population of cells defined by relatively high expression of CD11b, CD16, and HLA-DR, intermediate levels of CD14 and low levels of CD15 and CD33. Altogether, these data reveal the ability to discern unique cell populations with few cell surface markers within the PBMC compartment of patients with clinically localized ccRCC.

**Fig. 3.**
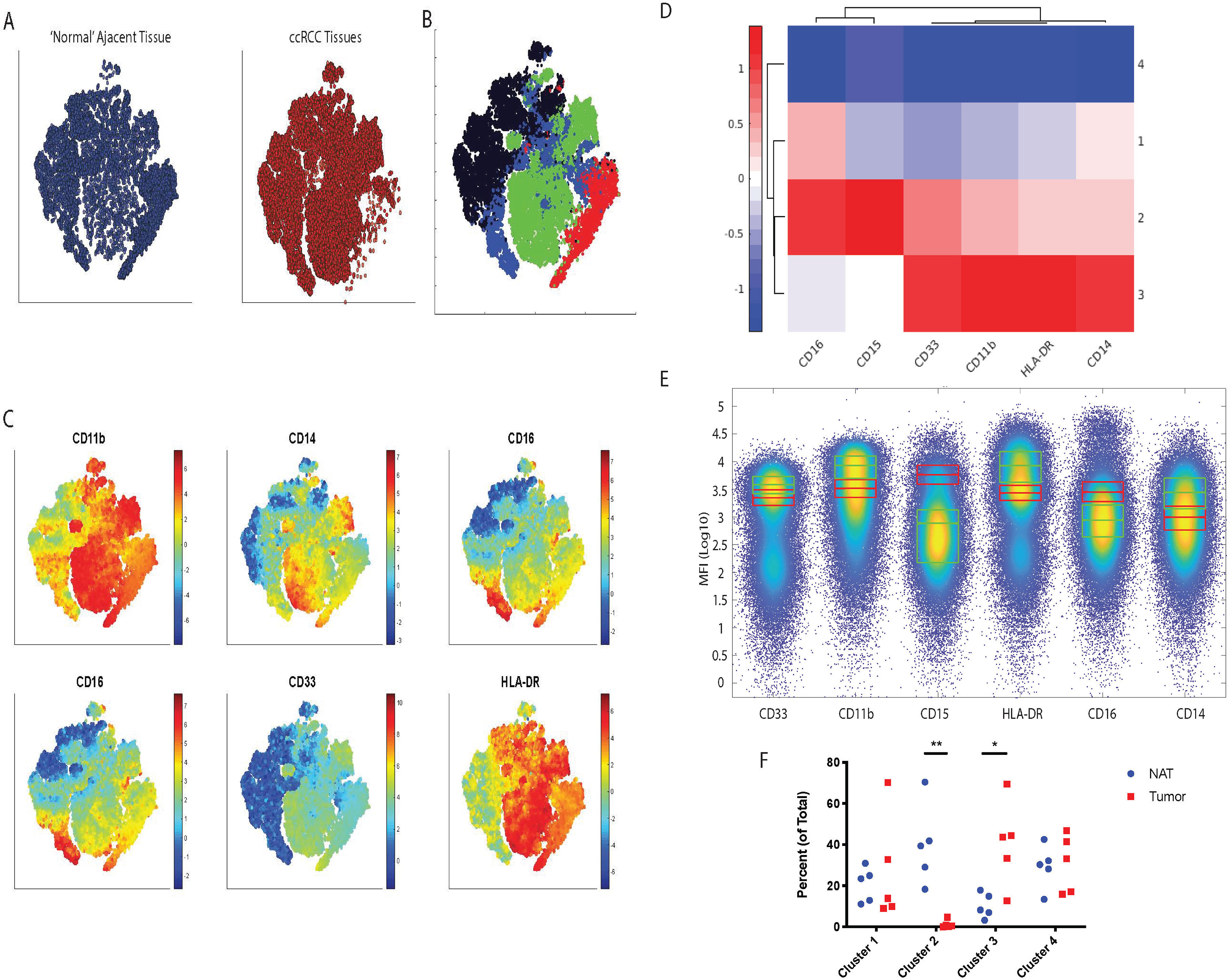
**ExCYT Analysis Reveals Different Cell Clusters Between the NAT and Tumor Tissue Compartments.** *A*, Pooled NAT (left, *blue*) and ccRCC tissues (right, *red*) depicted via t-SNE plots revealing high-level differences between tissue types. *B*, t-SNE plot of combined NAT and ccRCC flow cytometry data and clustering utilizing GMMs reveals 4 distinct cell population clusters; Cluster 1: Blue; Cluster 2: Red; Cluster 3: Green; Cluster 4: Black. *C*, t-SNE plots generated from Fig. 3*B* depicted as heat maps of various myeloid marker expression across combined NAT and ccRCC tissues indicating expression levels of the corresponding myeloid marker across both tissue types with red indicating higher levels of expression and blue indicating lower levels of expression. *D*, Interrogation of myeloid marker expression across the 4 distinct cell population clusters reveals profound differences between clusters 2 and 3 (*red* indicating higher levels of expression and *blue* indicating lower levels of expression). *E*, High-dimensional flow plots represent pooled NAT and tumor flow cytometry data with box-plots of clusters 2 (*red* boxes) and 3 (*green* boxes) demonstrate differences in terms of myeloid marker expression from flow cytometry data. *F*, Percent contribution of all 4 clusters within the NAT or ccRCC tumor tissues.

We next interrogated the NAT and Tumor flow cytometry data to identify unique signatures present in either tissue types. Using ExCYT (14), we identified 4 clusters present within the compiled data (Fig. 3*A*–3*B*; supplemental Fig. S1*B*). Interestingly, clusters 2 (red, enriched in NAT) and 3 (green, enriched in ccRCC) represented the clusters that were statistically significant and enriched in the NAT and ccRCC tissues, respectively (Fig. 3*C*–3*F*). Cluster 2 was defined by higher expression of CD15 and CD16, whereas cluster 3 was defined by relatively higher expression of CD11b, CD14, CD33, and HLA-DR. Taken together, these data provide evidence that the NAT may be characterized by CD15^+^CD16^+^ cells of potential granulocytic identity whereas the TME is characterized by CD11b^+^CD14^+^CD33^+^HLA-DR^+^ cells of potential monocytic/macrophage cellular identity.

##### Gene Expression Analysis Identifies Several Myeloid-Related Genes Are Up-regulated in Tumor Samples Relative to NAT

**Fig. 4.**
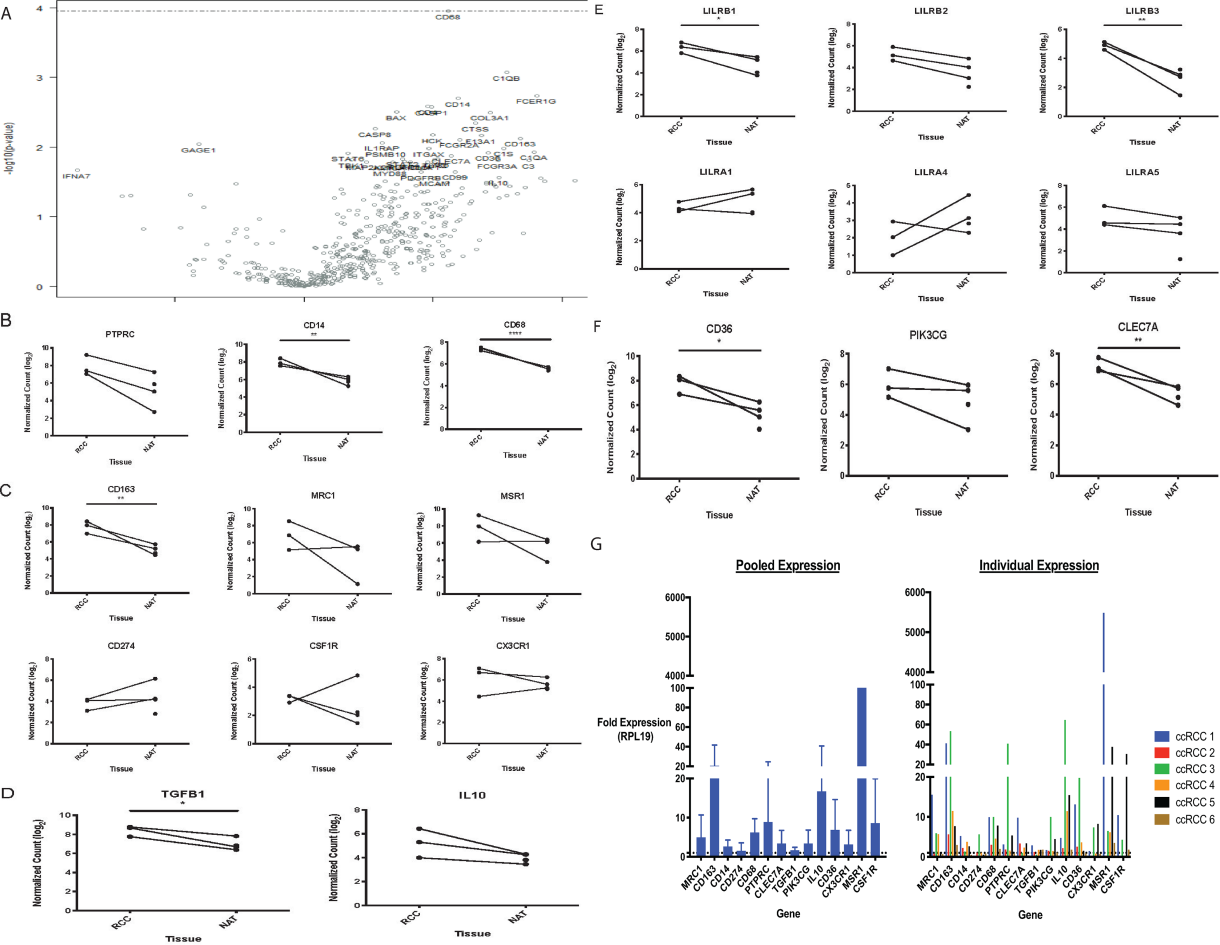
**Gene Expression Analysis via Nanostring Reveals Unique Genes Enriched in CD45^+^ Cells Isolated from ccRCC Tissues Relative to NAT.** *A*, Volcano plot representing differentially expressed genes between tumor CD45^+^ enriched fractions *versus* NAT CD45 enriched fractions. *B*, Normalized log2 count expression of lineage markers, *C*, various 'M2' macrophage markers, *D*, immunosuppressive factors, *E*, LILR family members, *F*, additional top differentially expressed genes. *G*, qRT-PCR analysis validates differentially expressed genes identified via NanoString. Left graph shows data where each sample was pooled, right graph demonstrates data for individual patient samples. Each ccRCC CD45^+^ enriched fraction data were normalized to patient-matched NAT CD45^+^ enriched fraction using the RPL19 housekeeping gene, *n* = 6 independent experiments.

To investigate differences between immune compartments within the NAT relative to the TME, we performed magnetic-bead based CD45^+^ cell enrichment of patient-matched NAT and tumor samples and subjected the samples to gene expression analysis using the NanoString Platform (Fig. 4*A*–4*H*, supplemental Table S2.) These data revealed differences in the immune infiltrate between the two tissue compartments, with expression of the macrophage marker *cd68* being among the highest differentially expressed genes, along with other known macrophage markers (Fig. 4*A*). Specifically, the tumor tissue expressed higher levels of *cd14* and *cd68* indicative of a greater presence of monocytes and macrophages in the tumor tissue compared with NAT (Fig. 4*B*). Furthermore, a number of markers of alternatively activated or 'M2' genes were expressed at higher levels within the tumor tissue compared with NAT such as *cd163, mrc1, msr1* whereas there were similar levels of PD-L1 (*cd274*) expression in the TME and NAT provided evidence that the TME harbors 'M2' or tumor-promoting macrophages (Fig. 4*C*). In addition, several immunosuppressive factors were expressed at greater levels in the TME relative to NAT and included *tgfb1*, and *il10* (Fig. 4*D*). Lastly, several novel targets were expressed at greater levels in the TME relative to NAT (Fig. 4*F*).

Given significant up-regulation of myeloid-related genes in CD45^+^ enriched fractions in the TME relative to patient-matched NAT, we sought to validate several top differentially expressed genes by quantitative Reverse Transcription Polymerase Chain Reaction (qRT-PCR). A total of 6 clinically localized ccRCC tissues and matched NAT underwent CD45^+^ enrichment as previously described (Fig. 4*G*). Expression of macrophage markers (*cd68*) along with 'M2' markers (*mrc1, cd163, cd36*) were up-regulated at the transcript level by qRT-PCR in ccRCC tissues relative to matched NAT. Furthermore, other macrophage genes were also up-regulated including *cx3cr1, msr1, csf1r*. The immunosuppressive cytokine *il10* was also up-regulated, providing evidence for a common mechanism of immune-evasion of a M2 macrophage phenotype. Altogether, these data confirm differential gene expression of myeloid-related genes in the tumor relative to NAT.

##### Proteomic Analysis of CD45^+^ and CD45^−^ Populations Derived from RCC and NAT

**Fig. 5.**
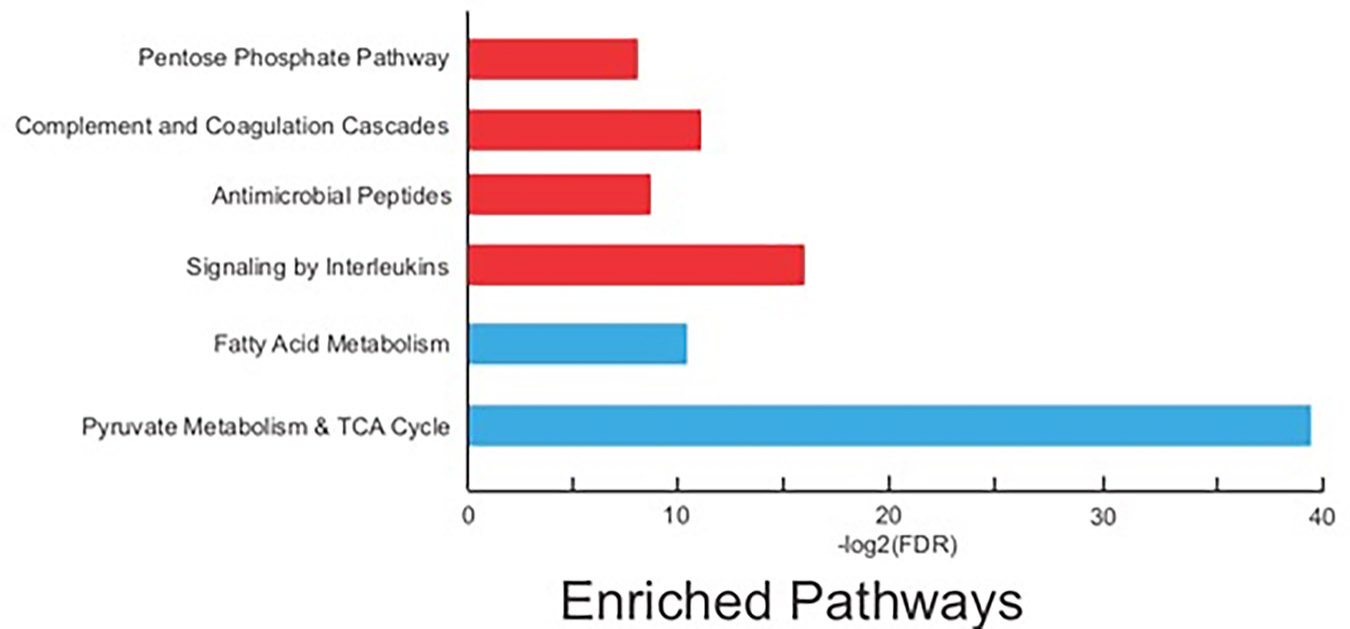
**Differential expression of protein profiles between CD45^+^ cell populations in normal adjacent tissues (NAT) and renal cell carcinoma (RCC) tissues.** Significantly up-regulated pathways (adjusted p < 0.05) are reported for CD45^+^ cell populations in RCC tissues (*red*) and CD45^+^ cell populations in NATs (*blue*).

Isolated cells (NAT-derived CD45^+^, NAT-derived CD45^−^, RCC-derived CD45^+^, RCC-derived CD45^−^, and PBMCs) from two patients were subjected to tryptic digestion and nano-LC–MS/MS analysis on an Orbitrap Lumos Fusion mass spectrometer. The combined spectra from all conditions resulted in the identification of 3929 protein groups (supplemental Table S3 - MaxQuant results table), with 3329 proteins quantified in at least one cell population from both patients. Next, we explored the differential expression of proteins between CD45^+^ cells derived from NAT and RCC samples. In total, 1049 proteins were quantified across CD45^+^ cells derived from RCC tumor and NAT samples, observing 595 differentially expressed proteins (log2 fold-change < 1.0). In CD45^+^ cells derived from RCC tissues, 353 proteins were increased in abundance relative to CD45^+^ cells derived from NATs, whereas 242 proteins were increased in abundance in CD45^+^-derived from NATs relative to CD45^+^ cells localized in tumor tissues. Pathway analysis of the differentially expressed proteins between NAT- and RCC tumor-derived CD45^+^ cells revealed the up-regulation of cellular processes associated with metabolism, including the TCA cycle, pyruvate metabolism, and fatty acid metabolism in NAT CD45^+^ cells (Fig. 5, supplemental Table S4 - **WebGestalt Pathway Analysis table**). In CD45^+^ cells derived from RCC tissues, pathways associated with activated immune processes were up-regulated, including interleukin signaling, complement and coagulation cascades, and antimicrobial peptide response, as well as a distinct metabolic profile (pentose phosphate up-regulation) compared with NAT-derived CD45^+^ cells. (Fig. 5, supplemental Table S4 - **WebGestalt Pathway Analysis table**). Interestingly, we observed several proteins robustly detected in RCC-derived CD45^+^ cells, but not NAT-derived CD45^+^ cells including CD36, CXCL8 and VAV1, further supporting a disparate activated immune profile associated with of CD45^+^ cells in RCC tissues relative to CD45^+^ cells in NATs.

## DISCUSSION

Immunotherapy has emerged as a fourth pillar of cancer treatment (19, 24, 25). However, beneficial responses in the setting of immunotherapy still represent the minority of outcomes while mechanisms or resistance to immunotherapy are being investigated. One such mechanism involves myeloid cells within the TME which directly initiate, promote tumor growth or create an immunosuppressive niche via a variety of different mechanisms (26–28). Burgeoning evidence highlights the efficacy of immunotherapy for ccRCC patients. The first Phase III trial (CheckMate 025) compared Nivolumab (anti-PD-1) therapy to everolimus in locally advanced or metastatic RCC and demonstrated an OS benefit and higher overall response rate for patients on nivolumab therapy (29). Additional strategies include recent attempts to combine multiple checkpoint inhibitors to further boost adaptive immune responses. Recent reports from the CheckMate 214 phase III trial revealed the combination of anti-CTLA-4 (ipilimumab) and Nivolumab resulted in significant overall survival benefit and higher overall response rates (30). These results are encouraging and provide proof that therapies targeting the immune system can generate clinically meaningful results. However, responses still represent the minority of patients and patients from these trials harbored advanced disease. Therapeutic intervention in the setting of clinically localized disease via strategies guided by knowledge of mechanisms of immune-evasion may afford additional benefit and may hopefully curtail the development of advanced disease. Thus, we sought to identify immune molecular underpinnings present in clinically localized disease that may provide insight into novel targets and therapies in ccRCC.

Using flow cytometric, unsupervised analytic approaches; our data provides further insight into the myeloid infiltrate in clinically localized ccRCC. We identified unique myeloid populations present in the blood of some patients but not others despite similar disease status. Given these differences, a limited combination of myeloid markers may prove informative with regards to patient prognosis. Furthermore, these data reveal differences in the cellular infiltrate within the TME and NAT. We also identified several differentially expressed myeloid genes in the TME relative to NAT, underscoring the role of myeloid cells within clinically localized ccRCC. These genes include markers associated with 'M2' macrophages as well as several genes of functional significance providing evidence of active immunosuppression in the TME (Fig. 4*D*). Interestingly, several members of the leukocyte immunoglobulin-like receptor (LIR) family were differentially expressed between TME and NAT. These proteins are involved in regulating inflammatory responses by myeloid cells and have opposing roles. The LILRB family possesses immunoreceptor tyrosine–based inhibitory motifs (ITIMs) and have been implicated in restraining inflammatory responses (31). Several LILRB family members (*lilrb1, lilrb2, lilrb3*) were expressed at higher levels within the TME relative to NAT. Conversely, activating members of the family, namely *lilra1* and *lilra4* were expressed at lower levels whereas *lilra5* was expressed at higher levels in the TME (Fig. 4*E*). These data provide evidence for different functions in different tissue compartments that may have biological implications. These targets may be specifically up-regulated in clinically localized ccRCC or may represent targets up-regulated in clinically localized cancers across tissue types. The expression level of these genes in advanced disease is also of interest given that they may continue to be viable targets.

Limitations of the current study include low sample sizes and the heterogeneity inherent in the outbred human population as well as the heterogeneity among macrophage infiltrate within solid tumors. Of note, density gradients used to obtain immune cells may result in loss of some cells given varying densities (32). Furthermore, multi-parameter flow cytometry may definitively identify individual cells expressing the various differentially expressed genes of interest. Gene expression analysis at a more granular level may resolve cell identity and expression patterns and will certainly prove informative. In our study, we determined that clinically localized ccRCC tumor samples are infiltrated by a variety of myeloid cells relative to NAT. Increased myeloid infiltration has been correlated with poor prognosis and shown to directly initiate tumorigenesis (26, 27). The genes identified revealed novel potential therapeutic targets that may promote immune responses in the setting of clinically localized ccRCC. Future studies may build upon this work to unravel the importance of these immune-suppressive mechanisms for tumor survival and to investigate whether therapeutic modulation of these targets *in vivo* may be of clinical benefit.

## Data Availability

All data has been uploaded to and the identifier is PXD014370. MaxQuant search results were uploaded to MS-Viewer (20) for viewing of annotated spectra with the search key dztdd1npww.

## Supplementary Material

Supplemental Table 1

Supplemental Table 2

Supplemental Figure 3

MaxQuant search results

Supplemental Data CD45+ NAT-Tumor Analysis
